# Temperature-dependent photo-elastic coefficient of silicon at 1550 nm

**DOI:** 10.1038/s41598-023-46819-0

**Published:** 2023-11-09

**Authors:** Johannes Dickmann, Jan Meyer, Mika Gaedtke, Stefanie Kroker

**Affiliations:** 1 Institute for Semiconductor Technology, Technical University of Braunschweig, Hans-Sommer-Str. 66, 38106 Brunswick, Germany; 2 Laboratory for Emerging Nanometrology, Langer Kamp 6a/b, 38106 Brunswick, Germany; 3 Physikalisch-Technische Bundesanstalt, Bundesallee 100, 38116 Brunswick, Germany

**Keywords:** Materials science, Materials for optics, Techniques and instrumentation, Optics and photonics, Optical materials and structures, Silicon photonics

## Abstract

This paper presents a study on the temperature dependent photo-elastic coefficient in single-crystal silicon with (100) and (110) orientations at a wavelength of 1550 nm. The measurement of the photo-elastic coefficient was performed using a polarimetric scheme across a wide temperature range from 5 to 300 K. The experimental setup employed high-sensitivity techniques and incorporated automatic beam path correction, ensuring precise and accurate determination of the coefficient’s values. The results show excellent agreement with previous measurements at room temperature, specifically yielding a value of $dn/d\sigma = -2.463 \times 10^{-11}$ 1/Pa for the (100) orientation. Interestingly, there is a significant difference in photo-elasticity between the different crystal orientations of approximately $50\%$. The photo-elastic coefficient’s absolute value increases by approximately 40% with decreasing temperature down to 5 K. These findings provide valuable insights into the photo-elastic properties of silicon and its behavior under varying mechanical stress, particularly relevant for optomechanical precision experiments like cryogenic gravitational wave detectors and microscale optomechanical quantum sensors.

## Introduction

Silicon is widely recognized for its extensive use as an optical material in both macroscopic and microscopic optomechanical applications^1–6^. While its indirect band gap of approximately 1.12 eV renders it unsuitable for visible spectrum applications due to high interband absorption, it exhibits low optical absorption in the near-infrared band beyond 1200 nm, making it highly advantageous for high-power applications. The prevalence of powerful and stable lasers operating at 1550 nm in fiber-optic communication further positions silicon as a favorable choice for optical components. Moreover, its low mechanical loss at cryogenic temperatures^7,8^ suggests its applicability in low thermal noise experiments such as gravitational wave detectors^9,10^ and laser frequency stabilization cavities^11,12^ and micro-scale optomechanical systems in the quantum regime. For these applications, the photoelasticity of silicon is a curse and blessing at the same time. High photoelastic coefficients typically go along with large optomechanical couplings. That can be exploited in measurement systems employing, for example, micro-scale oscillators^13^.

Additionally, however, statistical temperature fluctuations in optical components result in refractive index changes and subsequent phase fluctuations due to the influence of the photo-elastic coefficient. This phenomenon, known as photo-elastic noise, plays a critical role in limiting the sensitivity of high-precision opto-mechanical measurements such as future gravitational wave detectors as the Einstein Telescope or Cosmic Explorer^14–17^. To address these limitations, cooling the optical components to cryogenic temperatures presents a potential solution to mitigate both birefringence and photo-elastic noise.

In addition, applications involving massive optical components and high laser powers often encounter challenges associated with photo-elastic effects^15^. The substantial mass of these components induce significant mechanical stresses, which, in turn, generate a refractive index profile through the photoelastic coefficient $dn/d\sigma$, effectively creating a birefringent lens-like element. This profile can significantly impact the optical functionality of a setup and potentially lead to severe performance degradation.

**Figure 1:**
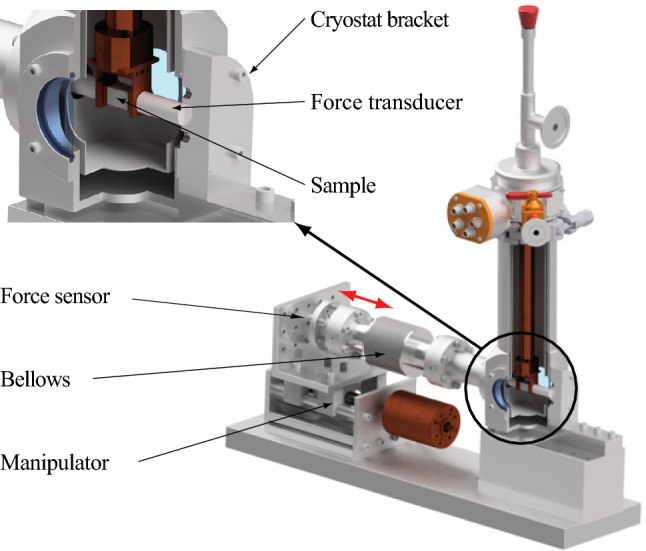
Computer-aided design (CAD) model of the cryostat used for the measurement of the photo-elastic coefficient. The CAD model showcases the specially designed high-force vacuum manipulator utilized in the experiment.

Unfortunately, the available literature on the photo-elastic coefficient only provides data at room temperature^18^, despite the majority of potential applications operating or being proposed for operation at temperatures below that threshold (Fig. 1).

## Results and discussion

This letter presents a measurement of the photo-elastic coefficient, $dn/d\sigma$, in single-crystal silicon across a wide temperature range of 5 K to 300 K. To perform this measurement, a polarimetric scheme was combined with a high-force vacuum manipulator specifically designed for cryogenic temperatures. This setup enabled precise and reproducible measurements of the refractive index change *dn* per applied mechanical stress $d\sigma$, with an uncertainty of $4\times 10^{-13}$ 1/Pa. The experiment comprises a polarimeter, which detects the polarization change of 45$^\circ$ linearly polarized light induced by mechanical stress in the sample. By analyzing this polarization change, the photo-elastic coefficient $dn/d\sigma$ can be determined.

**Figure 2:**
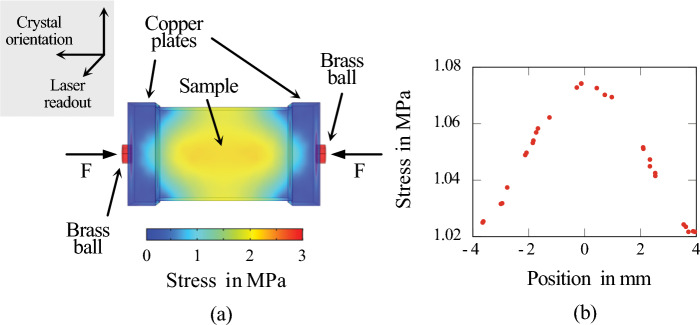
Finite element simulations were conducted using COMSOL Multiphysics to analyze the behavior of the mechanically constrained sample within the sample holder. The sample dimensions are $10\times 10\times 15$ mm$^3$. Figure (**a**) illustrates a cross-sectional view of the sample perpendicular to the optical axis of the readout laser beam. This view provides insight into the stress distribution within the sample for exemplary mechanical load. Figure (**b**) depicts a linear section taken vertically through the sample’s center of gravity.

To determine the photoelastic coefficient, first, generating a linear mechanical strain in the sample is necessary. To accomplish this, a sample holder developed (see Appendix A) using COMSOL Multiphysics simulations^19^. The results of this numerical study are depicted in Fig. 2. Using brass balls in the sample holder design ensures a highly uniform stress distribution at the center of the sample. Figure 2b shows a cross-section through the sample’s center, displaying the stress distribution. Notably, minor deviations at the sample center have negligible effects on the applied stress. However, in particular, for repetitive measurements, for example, in dependence on temperature, the laser beam position has to be monitored and readjusted to minimize measurement deviations induced by a drift of the position. Appendix B provides a detailed description of our system for automatic laser readjustment during the readout process. Through the implementation of this system, we achieve reduced measurement errors compared to all previously reported systems^18^.

**Figure 3:**
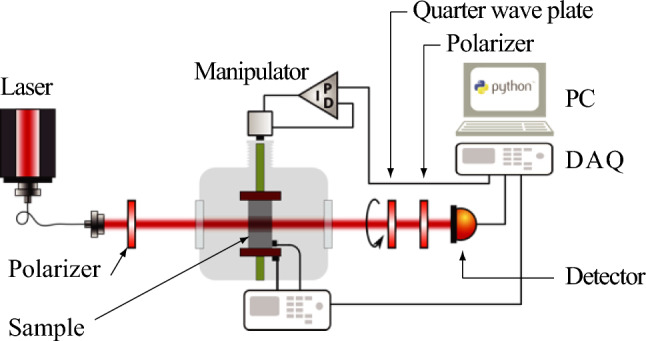
The experimental setup is represented by a simplified schematic. Initially, laser light emitted from the NKT Photonics Fianium SuperK source undergoes linear polarization by passing through a polarization filter (Thorlabs LPNIR100) set at an angle of $45^\circ$. The linearly polarized light then traverses the strained sample contained within the cryostat, resulting in a modification of the polarization due to photoelastic effects. Subsequently, the altered polarization state is detected and measured using a polarimeter (Thorlabs K10CR1/M with AQWP10M-1600 and LPNIR100) along with a photodetector (Thorlabs PDA50B2).

A schematic representation of the optical readout system is illustrated in Fig. 3. The system utilizes a laser system (NKT Photonics SuperK Fianium) that is initially linearly polarized using a polarization filter set at $+45^\circ$ (Thorlabs LPNIR100). After passing through the sample, a polarimeter detects the change in polarization state. The polarimeter comprises a combination of a rotating quarter wave plate (Thorlabs K10CR1/M with AQWP10M-1600) and a horizontal polarization filter (Thorlabs LPNIR100). To measure the light intensity, a photodetector (Thorlabs PDA50B2) and a DAQ card (National Instruments USB 6363) are employed, automatically recording the intensity for various angles of the $\lambda /4$ plate. It is important to note that the Mueller matrix of the polarimeter describing the polarization conversion solely depends on the angle $\psi$ between the quarter wave plate and the polarizer:

1 $$\begin{aligned} {\hat{M}}_\text {pol} = \begin{pmatrix} {1}/{2} &{} \cos ^2 (2\psi ) &{} -\sin (2\psi ) \cos (2\psi ) &{} \sin (2 \psi ) \\ {1}/{2} &{} \cos ^2 (2\psi ) &{} -\sin (2\psi ) \cos (2\psi ) &{} \sin (2 \psi ) \\ 0 &{} 0 &{} 0 &{} 0 \\ 0 &{} 0 &{} 0 &{} 0 \end{pmatrix}. \end{aligned}$$

Thus, the intensity $I(\psi )$ measured at the detector depends on the input Stokes vector $\vec {S}=(S_0,S_1,S_2,S_3)^T$ behind the sample:

2 $$\begin{aligned} I(\psi ) = \frac{1}{2} \left[ \left( S_0 + \frac{S_2}{2} \right) + S_3 \cos (2\psi ) +\frac{S_1}{2} \cos (4\psi ) -\frac{S_2}{2} \sin (4\psi ) \right] \end{aligned}$$

The angle-dependent intensity measured by the detector represents a truncated Fourier series. By performing Fourier analysis on the angle-dependent intensity measurements, the Fourier coefficients can be obtained. Through the photoelastic material properties, a mechanical stress introduces a phase shift between the horizontal and vertical components of the electric field, which can be quantified as $\Delta \varphi$. This phase shift can be determined from the Stokes vectors $S_3$ and $S_2$:

3 $$\begin{aligned} \Delta \varphi = \arctan \frac{S_3}{S_2}. \end{aligned}$$

**Figure 4:**
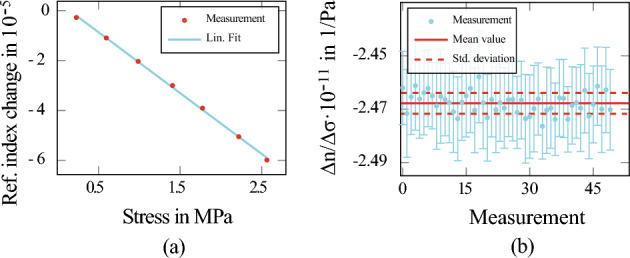
This figure shows the measurement of the photoelastic coefficient of silicon (100) at room temperature. In (**a**), the polarimetrically determined birefringence $\Delta n$ is presented for various levels of applied mechanical stresses $\sigma$. (**b**) Displays the photoelastic coefficients derived from the slope of the measurements, averaged over 50 repetitions.

By combining knowledge of the mechanical stress applied to the sample with polarimetric measurements of its birefringence, the photo-elastic coefficient can be determined as follows:

4 $$\begin{aligned} \frac{\Delta n}{\Delta \sigma } = \frac{\lambda }{2\pi L} \frac{\Delta \varphi }{\Delta \sigma }, \end{aligned}$$

where $L=10.00$ mm is the geometrical light path through the sample. The ratio $\Delta n/ \Delta \sigma$ is calculated by performing a linear regression analysis on the relationship between the birefringence $\Delta n$ and different mechanical stresses $\sigma$. Figure 4a displays one of these measurements conducted at room temperature. To ensure statistical reliability, this measurement was repeated 50 times at room temperature, allowing for the determination of a standard deviation for the measured values, compare Fig. 4b:

5 $$\begin{aligned} \frac{\Delta n}{\Delta \sigma } (T=293\,\text {K}, \text {Si}(100)) = (-2.463\pm 0.034)\times 10^{-11} \,{1}/{\text {Pa}}. \end{aligned}$$

The uncertainty associated with this measurement is more than a factor of three smaller than the uncertainty of the most precise literature value currently available^20^.

**Figure 5:**
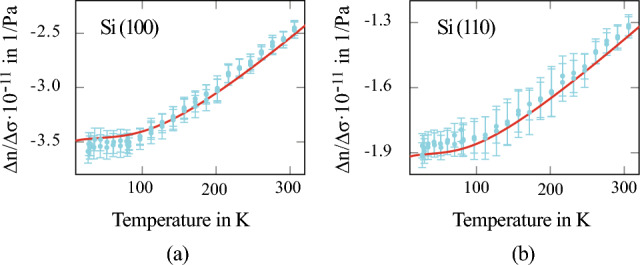
The final result of this study presents the photoelastic coefficient of silicon in the (100) and (110) directions across a temperature range of 5 K to 300 K. The measurement points are depicted in blue, while the phenomenological trend is shown in red, indicating the agreement between the experimental data and the fitted model.

The temperature-dependent measurement of the photoelastic coefficient was conducted using the cryostat (Janis ST-100, compare Fig. 1) in a temperature range of 5 K to 300 K. The temperature steps were set at 15 K intervals between 5 and 80 K (liquid helium temperatures) and 20 K intervals between 80 and 300 K (liquid nitrogen temperatures). The results of the measurements are presented in Figure 5 for two crystal orientations, Si(100) and Si(110). A significant change in photoelasticity is observed in both cases between 80 and 300 K, with the photoelastic coefficient becoming more prominent at cryogenic temperatures by approximately $40\%$. Notably, the photoelastic coefficient is approximately twice as strong in magnitude in the Si(100) direction compared to the Si(110) direction. To model the measurement results, a phenomenological relation based on the Kramers-Kronig relation for the photoelastic tensor was employed^21^:

6 $$\begin{aligned} \Gamma _{kl} = \left( 1 - \frac{1}{n_0^2} \right) ^2\frac{2D_{kl}}{\varepsilon _D} \left[ 1+K_{kl}\left( 1-\frac{\lambda _0^2}{\lambda ^2} \right) \right] , \end{aligned}$$

where $n_0$ is the unperturbed refractive index, $D_{kl}$ is the potential of deformation inside the sample, $\varepsilon _D$ is the dispersion energy, $K_{kl}$ are the disperion parameters of the crystal and $\lambda _0$ is the crystallographic position parameter of silicon. The photoelastic tensor $\Gamma _{kl}$ directly corresponds to the photo-elastic coefficient:

7 $$\begin{aligned} \Delta n_i = \frac{n_0^3}{2}\Gamma _{ij}C_{ij}^{-1}\sigma _j. \end{aligned}$$

In this equation, the Einstein summation convention is utilized. The tensor $C_{ij}$ denotes the elastic tensor of the sample:

8 $$\begin{aligned} {C}_{ij} = \begin{pmatrix} c_{11} &{} c_{12} &{} c_{12} &{} 0 &{} 0 &{} 0 \\ c_{12} &{} c_{11} &{} c_{12} &{} 0 &{} 0 &{} 0 \\ c_{12} &{} c_{12} &{} c_{11} &{} 0 &{} 0 &{} 0 \\ 0 &{} 0 &{} 0 &{} c_{44} &{} 0 &{} 0 \\ 0 &{} 0 &{} 0 &{} 0 &{} c_{44} &{} 0 \\ 0 &{} 0 &{} 0 &{} 0 &{} 0 &{} c_{44} \end{pmatrix}, \end{aligned}$$

where $c_{11} = 165.7$ GPa, $c_{12} = 63.9$ GPa and $c_{44} = 79.6$ GPa^22^.

To accurately model the temperature dependence of the photo-elastic coefficient, the temperature-dependent variations of the dispersion parameter *K* and the dispersion energy *D* are incorporated^23^. The mechanical properties, represented by the deformation potential *D*, exhibit minimal changes and can be considered as constant^23^. The refractive index of silicon, which is dependent on temperature and wavelength, is obtained from^24^. All parameters are presented in Appendix C in the supplementary file. For room temperature, $\Gamma _{11} - \Gamma _{12} = -0.126$ and $\Gamma _{44} = -0.053$. The values correspond to a ${\Delta n}/{\Delta \sigma } = -2.617 \times 10^{-11} {1}/{\text {Pa}}$ for silicon in (100) direction and ${\Delta n}/{\Delta \sigma } = -1.378 \times 10^{-11} {1}/{\text {Pa}}$ for silicon in (110) direction. Comparing the results to the values of the photo-elastic tensor presented in reference^20^ small deviations for the values of $\Gamma _{44}-\Gamma _{12}$ are evident. The tensor component $\Gamma _{44}$, instead, is in very good agreement. The deviations can, however, be explained by the measurement uncertainty chosen in reference^20^, which was only estimated but not measured. The measured data, depicted in blue, are compared to the temperature-dependent curve in Fig. 5a and b, illustrating an excellent agreement between the fitted photoelastic coefficient and the actual measurement.

## Conclusions

The collected data, along with their corresponding measurement errors, are available for access via the Dryad Digital Repository. The repository can be reached through the following link: 10.5061/dryad.s7h44j1cw. Additionally, the repository houses the fitted photoelasticity data, sampled at 1 K intervals across the range of 0 K to 300 K for both measured crystal orientations.

Recent investigations into the role of photoelasticity within the proposed gravitational wave detector, the Einstein Telescope, suggest challenges in meeting the specified requirements, even at room temperature^15^. In our present study, we have ascertained that silicon’s photoelasticity experiences an approximate 40% increase in magnitude as temperatures trend toward the cryogenic range. As components of the Einstein Telescope are projected to function within these cryogenic conditions using silicon (around 10 K)^16^, concerns arise regarding the photoelastic behavior of key optical interferometer components. The acquired data has now unlocked the potential for novel optimizations in the blueprint of the detector. Furthermore, the influence of photoelasticity on ongoing laser stabilization cavities could constitute a notable noise source^14^. For this application, the available material will also lay the foundation for future enhancements and improvements of measurement sensitivity.

The acquired data holds the potential to intricately inform the development of pioneering optomechanical experiments and components established on cryogenic silicon platforms. Significantly, the material parameters unveiled in this study provide a granular understanding, enabling the meticulous incorporation of photoelastic coupling—one of the foremost optomechanical coupling mechanisms—into the blueprinting of upcoming innovations.

### Supplementary Information

Supplementary Information.

## Data Availability

The entirety of the data from this study is accessible on the Dryad Digital Repository, accessible through the following link: 10.5061/dryad.s7h44j1cw.
