# Supplementary material for: Temperature-dependent photo-elastic coefficient of silicon at 1550 nm
Source: Sci Rep. 2023 Nov 9;13:19455. doi: 10.1038/s41598-023-46819-0 (PMC10636003; doi:10.1038/s41598-023-46819-0)
Supplement: Supplementary file 1 — Supplementary Information. [file 41598_2023_46819_MOESM1_ESM.pdf]

SUPPLEMENTARY MATERIAL: Temperature-dependent Photo-Elastic Coefficient of Silicon at 1550 nm

J. Dickmann,[1,2] J. Meyer,[1,2] M. Gaedtke,[1,2] and S. Kroker[1,2,3]

[1)] *Technical University of Braunschweig, Institute for Semiconductor Technology, Hans-Sommer-Str. 66, 38106 Braunschweig, Germany*

[2)] *Laboratory for Emerging Nanometrology, Langer Kamp 6a/b, 38106 Braunschweig, Germany*

[3)] *Physikalisch-Technische Bundesanstalt, Bundesallee 100, 38116 Braunschweig, Germany*

(*Electronic mail: j.dickmann@tu-braunschweig.de)

(Dated: 27 October 2023)

Appendix A: Cryogenic sample holder

The development and fabrication of a dedicated sample holder were necessary to facilitate the temperature-dependent analysis of photoelasticity. This holder needed to fulfill dual objectives: 1. Uniform and symmetrical imposition of forces across the sample. 2. Efficient cooling of the sample to cryogenic temperatures via the holder. The ultimate configuration of this holder is illustrated in Figure 6. Our selection consists of a dual cantilever design crafted from copper, chosen for its remarkable thermal conductivity. Enhancing structural connectivity, the sample is affixed to the cantilevers through two supplementary copper plates joined by brass balls. The cooling cantilevers are then attached to the cryostat's cooling finger. This cooling mechanism uses liquid nitrogen or helium to reduce the finger's temperature from ambient conditions to approximately 5 K. Thermally insulating rods are employed to administer the force to the sample, aided by a vacuum manipulator (refer to Fig. 1 in the primary section).

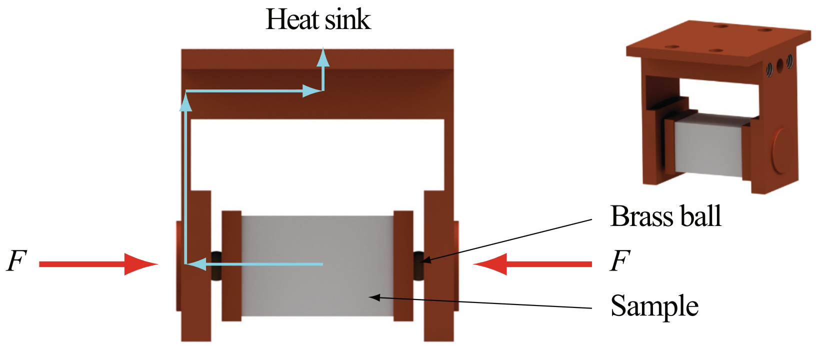

FIG. 6. Computer-aided design (CAD) model of the cryogenic sample holder designed for high-force vacuum manipulation. The red arrows indicate the manipulating forces applied to the sample, while the blue arrows represent the heat flow from the sample to the heat sink.

Appendix B: Temperature-dependent readjustment

The assessment of silicon sample photoelasticity was executed across a temperature spectrum ranging from 300 K to 5 K. Within this wide temperature span, the copper cooling finger of the cryostat underwent substantial length variations. Consequently, the assurance of the polarimeter's laser measuring the sample's birefringence at a precise position would be compromised. Given the anisotropic nature of the sample's stress distribution (as demonstrated in Fig. 2 of the main section), this potential misalignment held the potential to distort the photoelasticity measurement. To

counter this, another laser for alignment was integrated into the setup (1 mW, 520 nm laser diode - Thorlabs PL201). This alignment laser was adjusted to emit parallel to the primary measuring laser (wavelength 1.55 µm) onto the sample, as depicted in Fig. 7. Positioned at half the sample's height (equivalent to 5 mm) below the measuring laser, the alignment laser's intensity that fell below the lower edge was the only portion of its light capable of passing the non-transmissive silicon sample in the visual spectrum. The transmitted intensity was then separated from the measuring laser using a D-shaped mirror and then measured via a photodetector positioned behind the cryostat, as indicated in Fig. 7.

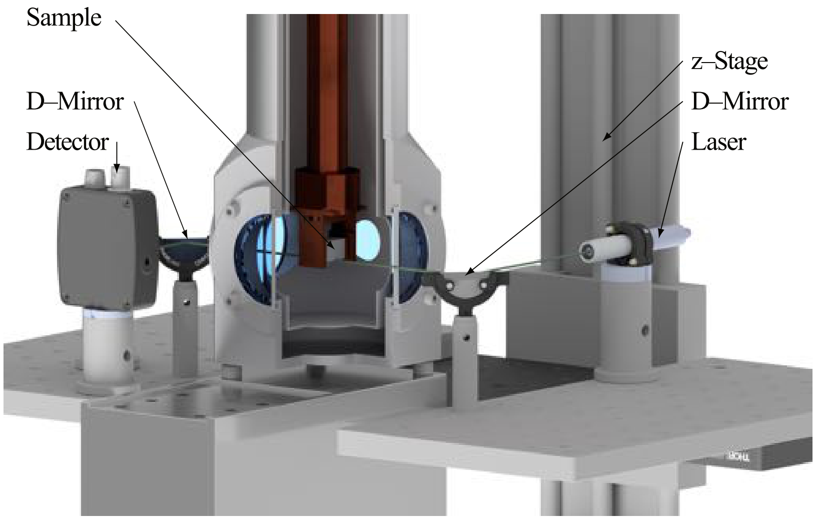

FIG. 7. Computer-aided design (CAD) model of the cryostat featuring a temperature-dependent readjustment system for the optical readout using an alignment laser. The CAD model highlights the components and mechanisms involved in the precise realignment of the optical readout caused by temperature variations.

Alterations in the sample's height due to temperature-induced shifts in the cooling finger led to concomitant variations in the transmitted intensity of the alignment laser. Since the alignment laser had a Gaussian intensity profile, these intensity fluctuations took the form of an error function. Figure 8(a) presents the recorded alignment laser intensity change during manual adjustments of the sample height. The fitted error function exhibited a near-zero fit error, underscoring the alignment laser's well-defined Gaussian shape. Furthermore, Fig. 8(a) displays the Gaussian profile, derived through numerical integration of the measured values. To enable consistent tracking of the measurement laser's height after each temperature shift within the cryostat, the complete optics of the polarimeter and alignment laser were mounted on a linear stage. This stage was manipulated via a stepper motor, delivering a vertical axis resolution surpassing 1 µm. The procedure depicted in Fig.

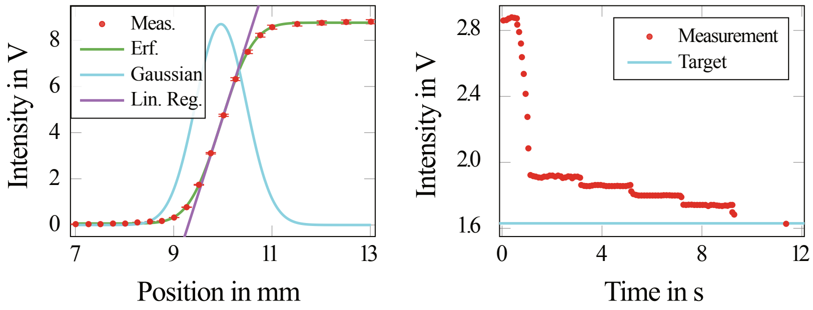

(a) (b)

FIG. 8. (a) The plot depicts the intensity of the detector as a function of the edge position. The red dots represent the measured data points. The green curve corresponds to the regression based on the error function, while the blue curve represents the Gaussian profile of the laser beam. (b) The graph displays the detector voltage for the readjustment plotted versus time.

8(b) outlines the progression toward the central position, depicting the measured alignment laser intensity versus time. Through this automated method, precise realignment of the measuring laser after every temperature alteration was attained. This process ensures that the photoelasticity within the sample's center is measured while reliably considering the calculated sample stress. In Fig. 8(b), it is evident that the realignment procedure can discern each incremental step of the stepper motor. Notably, each step corresponds to a minute height variation in the optical path, equivalent to 1 µm. This residual height deviation can be translated into a maximal stress error within the sample. To compute this, we refer to the assessment of height-dependent stress within the sample, as depicted in Fig. 2(b) in the main section of the paper. The residual height error results in a stress discrepancy of less than 0.05 %. To provide context for this outcome, it's important to note that without the optical realignment procedure described here, the stress error traverses well beyond 5 % across the entire temperature range from 5 K to 300 K. Consequently, the implemented realignment procedure demonstrates remarkable measurement precision of photoelasticity.

Appendix C: Silicon material parameters for the calculation of photoelasticity

The material parameters used for the calculation of temperature-dependent photo-elasticity are listed in Tab. 1 and Fig. 9.

TABLE I. Silicon material parameters for the calculation of photoelasticity [1,2].

**Parameter**			**Si(100)**	**Si(110)**
Deformation potential	D	[eV]	-1.25	-1.99
Oscillator wavelength	λ_0	[µm]	0.31	0.31
Wavelength	λ	[µm]	1.55	1.55

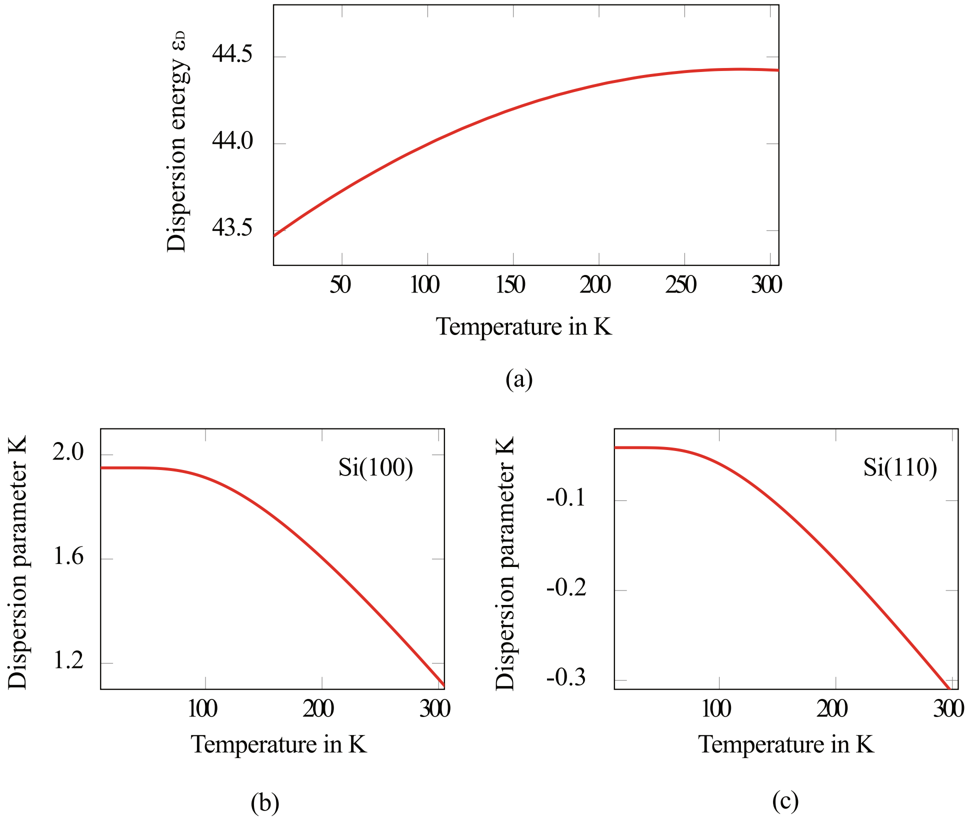

FIG. 9. Silicon material parameters for the calculation of photoelasticity [1,2]

[1] S. H. Wemple und W. DiDomenico. „Optical Dispersion and the Structure of Solids“. Phys. Rev. Lett. 23.20 (1969), 1156–1160.

[2] C. W. Higginbotham, M. Cardona und F. H. Pollak. „Intrinsic Piezobirefringence of Ge, Si, and GaAs“. Phys. Rev. 184.3 (1969), 821–829.
